# Characteristics, quality of life and control of respiratory allergic diseases caused by house dust mites (HDMs) in Spain: a cross-sectional study

**DOI:** 10.1186/s13601-019-0276-5

**Published:** 2019-08-06

**Authors:** Beristain Ana, Fernando de la Torre, the EpidMite Study Group, María Teresa Aldunate, José Antonio Álvarez, Miguel Añó, Gonzalo Campos, Victoria Cardona, Sergia Cruz, Virginia De Luque, Joan Domenech, Elena Escudero, Agustín Fernández, Javier Figueroa, Pere Gaig, Belén Gómez, Genoveva González, Diego Gutiérrez, Javier Iglesias, Vicente Jover, Raquel Kraemer, Tania Linares, Damián López, Sara López Freire, Estrella Llamas, Alfons Malet, Antonio Maraví, Joaquín Martín-Lázaro, Montserrat Molina, Javier Montoro, Carmen Moreno, Carmen Moya, Rafael Pamies, Laura Pérez-Pastor, Dolores Quiñones, Isabella Raducán, Fernando Rodríguez, Ramón Rodríguez-Pacheco, Idoia Rodríguez-Zuazo, María Rueda, Cesárea Sánchez, Inmaculada Sánchez-Guerrero, Inmaculada Sánchez-Machín, Carmen Vidal, Juan José Zapata

**Affiliations:** 1 H. Monte Naranco, Oviedo, Asturias Spain; 2 ALK-Abelló, S.A., C/Miguel Fleta 19, 28039 Madrid, Spain

**Keywords:** Allergic asthma, Asthma control, Allergic rhinitis, House dust mites, Pharmacologic costs, Quality of life

## Abstract

This multicentre, cross-sectional study conducted in Spain assessed the clinical characteristics and quality of life of patients who were aged 14–55 years and had allergic rhinitis and/or asthma, which was due to house dust mite sensitisation, for at least 2 years. Overall, 296 patients were included; 60% had allergic rhinitis (mostly persistent moderate-to-severe) and 40% had rhinitis and asthma (mostly intermittent or mild-to-persistent). Patients with rhinitis had moderately reduced quality of life, which was significantly worse in adults than adolescents. The impact of asthma on quality of life was less pronounced than that of rhinitis. Our findings show that allergic asthma and/or rhinitis due to house dust mites have a moderately negative impact on quality of life, particularly in adults, and that quality of life is significantly influenced by rhinitis.

The prevalence of house dust mite (HDM) sensitisation is high [1, 2] and represents a significant risk factor in the development of respiratory allergic disease [3–5]. Therefore, we conducted a multicentre, epidemiological, ambispective, cross-sectional study to determine the clinical characteristics of patients with HDM-induced respiratory allergic disease in Spain, along with their quality of life (QoL). A secondary objective was to assess the extent of asthma control during the year prior to inclusion in the study. Patients (aged 14–55 years) from 33 clinical groups working in Spain were eligible to participate if they had respiratory allergic disease (rhinitis or rhinitis and asthma with/without conjunctivitis) for at least 2 years that was due to HDM, had a positive skin prick test (wheal diameter ≥ 3 mm) and/or were immunoglobulin E positive (class 2 or above), as measured by ImmunoCAP (Thermofisher, Uppsala, Sweden), and who had not received immunotherapy in the 5 years prior to their inclusion in the study. The patients were included between October 2015 and February 2017, and gave written consent to participate in the study.

Rhinitis was assessed according to the Allergic Rhinitis and its Impact on Asthma (ARIA) guidelines [6] and asthma was assessed according to the Spanish Guideline on the Management of Asthma (GEMA) [7] and the Asthma Control Questionnaire (ACQ, Spanish adult version for all patients). Quality of life in patients with asthma was assessed by the mini version of the Asthma Questionnaire on Quality of Life (miniAQLQ). For rhinoconjunctivitis, QoL was determined using the mini Rhinoconjunctivitis Quality of Life Questionnaire (miniRQLQ) and the ESPRINT-15 [8]. Patients were asked about medication used for the symptomatic treatment of respiratory allergic disease caused by HDM in the year prior to their inclusion in the study. All information was collected during a single visit.

Overall, 296 patients (mean age of 29.5 ± 10.0 years) were evaluated (Table 1). Most patients (n = 179; 60.5%) had rhinitis without asthma and 117 patients (39.5%) had rhinitis with asthma. Almost all patients (98.7%) were sensitised to *Dermatophagoides pteronyssinus* and 78.3% of patients were sensitised to *D. farinae*. With respect to storage mites, 48.5% of patients tested positive for sensitivity to *Lepidoglyphus*, 31.5% for *Tyrophagus*, 20.3% for *Glycyphagus* and 15.5% for *Blomia*.

**Table 1:** Baseline characteristics

	Patients with rhinitis (without asthma)		Patients with rhinitis (with asthma)	
	n	%	n	%
Age group				
Adolescents (14–17 years)	20	11.2	16	13.7
Adults (18–55 years)	158	88.3	101	86.3
Gender				
Female	94	52.8	77	65.8
Male	84	47.2	40	34.2
Conjunctivitis				
Yes	114	64.4	74	63.3
Rhinitis				
Intermittent	40	22.6	27	24.3
Persistent	137	77.4	84	75.7
Mild	48	27.1	29	25.7
Moderate-to-Severe	129	72.9	84	74.3
Asthma				
Intermittent			47	41.2
Mild persistent			45	39.5
Moderate persistent			20	17.5
Severe persistent			2	1.8

In the year prior to inclusion, 58.8% of patients used allergen avoidance measures; 10.3% of patients visited the emergency room due to their allergy. More patients with asthma (20.7%) than with rhinitis (3.4%) had an emergency room visit because of their respiratory allergic disease in the year prior to inclusion. Only one patient required hospitalisation due to asthma during this period prior to inclusion.

The ESPRINT-15 questionnaire was used to evaluate the overall QoL in patients with rhinoconjunctivitis, as well as specific domains including Symptoms, Daily life activities, Sleep, Psychological effects and General state of health (Table 2). No statistically significant differences were seen among patients with rhinitis and asthma compared with those who had rhinitis alone (with/without conjunctivitis). There was a significant difference between adolescents and adults in the domains of sleep, psychological effects, general state of health and overall QoL, with a greater impact in the adult population (Table 2).

**Table 2:** Results of the Quality of Life Questionnaires

	Overall	Adolescents	Adults
ESPRINT-15 Quality of Life Questionnaire			
Symptoms (items 1–5)			
Mean (SD)	3.03 (1.41)	2.62 (1.45)	3.09 (1.40)
Median [Q1, Q3]	3.00 [2.00, 4.00]	2.20 [1.50, 3.90]	3.20 [2.00, 4.20]
		0.0635	
Daily life activities (items 6–8)			
Mean (SD)	2.23 (1.56)	1.85 (1.30)	2.28 (1.59)
Median [Q1, Q3]	2.33 [1.00, 3.33]	2.00 [0.67, 3.00]	2.33 [1.00, 3.33]
		0.1978	
Sleep (items 9–11)			
Mean (SD)	2.29 (1.83)	1.71 (1.72)	2.38 (1.84)
Median [Q1, Q3]	2.00 [0.67, 3.67]	1.33 [0.17, 2.83]	2.00 [0.67, 3.67]
		0.0338	
Psychological effects (items 12–14)			
Mean (SD)	2.38 (1.70)	1.82 (1.52)	2.47 (1.72)
Median [Q1, Q3]	2.33 [1.00, 3.67]	1.67 [0.50, 2.83]	2.33 [1.00, 3.67]
		0.0381	
General State of Health (item 15)			
Mean (SD)	3.08 (0.94)	2.72 (1.06)	3.13 (0.91)
Median [Q1, Q3]	3.00 [2.00, 4.00]	3.00 [2.00, 3.50]	3.00 [3.00, 4.00]
		0.0323	
Overall (items 1–15)			
Mean (SD)	2.57 (1.33)	2.13 (1.22)	2.63 (1.34)
Median [Q1, Q3]	2.53 [1.47, 3.47]	2.10 [1.23, 2.77]	2.60 [1.53, 3.53]
		0.0365	
MiniRQLQ Questionnaire			
Activities (items 1–3)			
Mean (SD)	2.66 (1.43)	1.94 (1.28)	2.76 (1.42)
Median [Q1, Q3]	2.67 [1.67, 3.67]	1.67 [1.00, 2.67]	2.67 [1.83, 3.67]
		0.0014	
Practical problems (items 4–5)			
Mean (SD)	3.77 (1.61)	3.42 (1.71)	3.82 (1.60)
Median [Q1, Q3]	4.00 [2.50, 5.00]	3.50 [2.00, 4.75]	4.00 [2.50, 5.00]
		0.1730	
Nasal symptoms (items 6–8)			
Mean (SD)	3.54 (1.48)	3.24 (1.55)	3.58 (1.47)
Median [Q1, Q3]	3.67 [2.67, 4.67]	3.33 [2.00, 4.00]	3.67 [2.67, 4.67]
		0.1988	
Ocular symptoms (items 9–11)			
Mean (SD)	2.15 (1.74)	1.81 (1.71)	2.20 (1.74)
Median [Q1, Q3]	2.00 [0.67, 3.33]	1.33 [0.17, 3.67]	2.00 [0.67, 3.33]
		0.2071	
Other symptoms (item 12–14)			
Mean (SD)	1.56 (1.71)	1.56 (1.71)	2.13 (1.59)
Median [Q1, Q3]	2.00 [0.67, 3.33]	1.00 [0.17, 2.33]	2.00 [0.67, 3.33]
		0.0242	
Overall (items 1–14)			
Mean (SD)	2.76 (1.29)	2.32 (1.32)	2.83 (1.28)
Median [Q1, Q3]	2.71 [1.79, 3.64]	2.00 [1.25, 3.57]	2.71 [1.86, 3.64]
		0.0386	

*SD* standard deviation

There was a significant difference in the miniRQLQ scores between adolescents and adults in the activities domain, in other symptoms and in the overall score (Table 2). There were no differences between patients who had asthma in addition to rhinitis compared with those who only had rhinitis (with/without conjunctivitis). Asthma was associated with a lower impact on QoL compared with rhinitis (medians of different domains: 4.7–5.8), with no statistically significant differences recorded between adolescents and adults. Patients had varying degrees of asthma control at baseline, as determined using ACQ scores: 45.2% of patients scored > 1.5 (inadequate control of the disease), 34.5% had adequate control (score < 0.75) and the remaining 20.2% had partially controlled asthma (0.75–1.5).

Having persistent severe asthma with additional conjunctivitis were the factors significantly associated with a worse result in the ACQ (higher score), in both the linear and the logistic model. Factors which reduced QoL in patients with rhinitis as measured by ESPRINT-15 and miniRQLQ included persistent, moderate-to-severe rhinitis with additional conjunctivitis and being female. For patients with asthma, adequately controlled asthma and absence of concurrent conjunctivitis were associated with improved quality of life.

This cross-sectional study of patients allergic to HDM in Spain indicates that respiratory allergic disease due to HDM has a moderate impact on patient QoL and this impact is influenced more by the presence of rhinitis than asthma. In some studies, sleep and daily activities were the QoL domains most affected in patients allergic to HDM [9, 10], but the current study found no differences between the different domains of rhinitis questionnaires. Several non-specific triggers can influence the severity of symptoms of respiratory allergic diseases, including changes in temperature, air pollution and cigarette smoke [11]. Variation in these factors could have been responsible for the differences in results between the present and previous studies. In the present study, differences between adults and adolescents were found in different domains of ESPRINT-15 and miniRLQL, indicating that both questionnaires provide complementary information.

Notwithstanding the limitations inherent in a cross-sectional study design, this study shows that Spanish patients with allergic asthma and/or rhinitis due to HDM have moderately reduced QoL, which is significantly influenced by rhinitis, despite continuous treatment. Age is a determining factor in QoL in this population, and its impact is more negative in adults than in adolescents.

These findings highlight the importance of effective management of upper airway symptoms in patients with respiratory allergic disease.

## Data Availability

The datasets used and/or analysed during the current study are available from the corresponding author on reasonable request.

## Abbreviations

ACQ: Asthma Control Questionnaire
CI: confidence interval
GEMA: Guía Española del Manejo del Asma [Spanish Guideline on the Management of Asthma]
HDMs: house dust mites
miniAQLQ: mini Asthma Questionnaire on Quality of Life
miniRQLQ: mini Rhinoconjunctivitis Quality of Life Questionnaire
OR: odds ratio
Q1: 1st quartile
Q3: 3rd quartile
QoL: quality of life
SD: standard deviation
